# A conceptual model of nurses’ workplace social capital: a theory synthesis

**DOI:** 10.1186/s12912-021-00660-w

**Published:** 2021-08-17

**Authors:** Jiamin Xu, Azadeh T. Stark

**Affiliations:** 1 Department of Nursing Sciences, Faculty of Medicine and Health, Lishui University, Lishui, China; 2 Department of Pathology and Laboratory Medicine, Henry Ford Health System, Detroit, MI USA; 3 School of Interdisciplinary Studies, University of Texas at Dallas, Richardson, TX USA

**Keywords:** Conceptual model, Nurses, Social capital, Theory synthesis, Workplace

## Abstract

**Background:**

Research has confirmed the importance of workplace social capital in the nursing workforce. Integration of the empirical evidence about nurses’ workplace social capital into a scientific collection can provide a comprehensive presentation of this concept. This scientific collection can be a conduit for further research and advancement of nursing management and leadership. The purpose of this paper, therefore, is to discuss the process of developing a conceptual model of nurses’ workplace social capital, an effective and concise approach to illustrate a scientific phenomenon.

**Methods:**

The model of nurses’ workplace social capital was developed following Walker and Avant’s strategy of theory synthesis. Empirical evidence relevant to nurses’ workplace social capital was synthesized by systematically examining the existing literature. PubMed, CINAHL, Web of Science and Google Scholar were searched periodically from October 2017 to July 2020.

**Results:**

Our proposed conceptual model lays out the determinants and outcomes of nurses’ workplace social capital and specifies the relational statements among these concepts. Nurses’ workplace social capital is influenced by the organizational and individual determinants shaped by multiple layers of sub-concepts. The development and implementation of nurses’ workplace social capital has three themes of consequences: 1) nurses’ outcomes; 2) patients’ outcomes; and 3) organizational outcomes. All the concepts and statements have been organized and aligned with the principles of “inventory of determinants or results” and “theoretical blocks”.

**Conclusion:**

Our theoretical synthesis offers a comprehensive picture of the current knowledge of nurses’ workplace social capital. Efforts should be dedicated to evaluating, revising, and revamping this newly developed model based on future empirical evidence. Our synthesized conceptual model is the segue to more comprehensive studies about nurses’ workplace social capital. Interventional programs for the development of social capital can be structured based on the identified determinants.

## Introduction

The healthcare industry is a complex and yet an adaptive system [1]. Nurses comprise the majority of healthcare professionals in any healthcare organization and weave their relational networks in their organizations through their interactions among themselves and with the other healthcare providers. These relational networks have been described as “workplace social capital” or “organizational social capital”. The global intensification of work-life and the importance of the quality of work environment on the workforce have put an unprecedented attention on workplace social capital [2].

Research portends the positive and promising influences of nurses’ workplace social capital [3, 4]. The value of findings from single studies is nil if the knowledge gained is not integrated into a network to present a more comprehensive understanding of the reported phenomenon [5, 6]. Despite extensive single studies on the concept of nurses’ workplace social capital, there is a lack of an evidence-based on its comprehensive presentation. A conceptual model (a graphic form of theory), that is based on the synthesis of previous work provides an effective way to depict and to develop a deeper understanding of the nurses’ workplace social capital [6].

## Background

The term “social capital” was originated from the domain of sociology and is regarded as an important element for organizational success through the networks of relationships [7]. Several social scientists have made significant contributions to the development of social capital; however, Bourdieu was the first who formally defined this concept in his 1986 publication entitled “*The forms of capital”*. He defined the concept of social capital as “the aggregate of the actual or potential resources which are linked to possession of a durable network of more or less institutionalized relationships of mutual acquaintance and recognition - or in other words, to membership in a group” [8]. About a decade later, Putnam introduced the notion of social capital to the field of empirical studies [9, 10]. Various dimensions with shared attributes have been proposed by scholars based on their interests in different facets of this phenomenon [11].

In the field of nursing, Read [12] **first coined the term “nurses’ workplace social capital”**.

Xu et al. [4] expanded on this concept to capture the contemporary changes, e.g. diversity in the demographic structure of the nursing workforce, or the assumption of more autonomy in the delivery of healthcare services by the nursing professionals. Meanwhile, several other nursing scholars have contributed to the development of social capital theories in the nursing profession. For instance, DiCicco-Bloom et al. (2007) constructed a “Social Capital Model” to facilitate understanding of the complex relationships in the operation of primary care work environment [1]. In 2013, Hofmeyer proposed a “Social Capital Framework”, with the objective of illustrating the importance of developing team relational networks in effective nursing management [13]. Gilbert et al. (2017) introduced the “Conceptual Model of Organizational Intellectual Capital”; this conceptual model further cements the vital position of social capital in nursing leadership [14]. Development of these theories and conceptual models of social capital in the nursing field mostly have been based either on resources borrowed from outside disciplines or limited nursing insights. Research supports the considerable influence of nurses’ workplace social capital on nurses’ mental and physical well-being, quality and efficiency of patient management and the overall healthcare organizations [4, 15–17]. Yet, so far, no theoretical model rooted in the integrative evidence of nursing studies that addresses workplace social capital has been developed. The aim of this paper is to report on the development of an evidence-based conceptual model of nurses’ workplace social capital, grounded on a logical theory synthesis approach, which can be implemented as a practical framework for future research and practice.

## Methods

The approach adopted to developing this model is based on the theory synthesis proposed by Walker and Avant [6]. This strategy enables to transform the results of empirical studies about a phenomenon of interest into an integrated whole; in other words, it brings the pieces of knowledge together in a logical way to form a more useful and coherent presentation. It is considered a step beyond the concept analysis and a specified approach for development of a theory. The process of theory synthesis consists of three iterative steps: specifying the focal concept(s), identifying factors related to the focal concept(s) and their relationships, and constructing an integrated presentation [6].

Theory synthesis is grounded in empirical evidence. In this paper, literature served as the source for synthesis of the theory in which the notion of nurses’ workplace social capita was grounded. The databases of PubMed, CINAHL, Web of Science and Google Scholar platform were searched, without time limitation, to identify relevant publications. Search strategies were developed based on the combinations of search terms “social capital”, “nursing”, “nurses” and “nurs*” with proper Boolean operators. For example, the strategy that was implemented in searching the PubMed consisted of the following steps: 1) # 1: social capital [MeSH] OR “social capital” [Title/Abstract]; 2) # 2: nursing [MeSH] OR nurses [MeSH] OR nurs* [Title/Abstract]; and 3) #3: # 1 AND # 2.

The initial search was conducted in October 2017 and the literature was periodically reviewed to capture the most current information. The final search, conducted in July 2020, was limited to full-text English articles in peer-reviewed journals. A total of 179 articles in PubMed, 506 in CINAHL, 185 in Web of Science and 75 in Google Scholar were identified and downloaded into the EndNote (version X8), and in consequence, 84 duplicate articles were eliminated. The remaining 861 retrieved publications were scanned by their titles and abstracts and studies were excluded (*n* = 524) if their objectives and/or scopes had excluded the concept of social capital. The remaining 337 articles then were reviewed for their contexts. Commentary papers and studies relevant to patients’ or nursing students’ social capital and social capital in scientific domains other than the nursing field were further excluded in this step. A total of 29 articles discussing quantitative, qualitative, and theoretical aspects of nurses’ workplace social capital were selected to draw evidence for the development of the model. Finally, the PhD dissertation work of the lead author exploring factors influencing nurses’ workplace social capital was included in the evidence pool of empirical research [6, 18]. The characteristics of the included studies are provided in Table 1.

**Table 1:** Characteristics of studies that were reviewed and included in the current conceptual model

Reference	Design	Sample/sources	Findings related to the current conceptual model	Main contribution to the current conceptual model*
Hafeez et al., 2020 [3]	Qualitative study	Hospitals nurses *n* = 20	Nurses perceived workplace social capital as a major strategy for prevention of occupational injuries and accidents	Identify nurses’ outcomes and propose relational statements
Jakobsen et al., 2020 [19]	Cluster randomized controlled trial	Nurse and nurse aids from five hospitals *n* = 625	The positive effects of organizational intervention of participatory workshops on utilizing assistive devices in patient handling on workplace social capital	Describe determinants (block 1) and propose relational statements
Xu, 2020 [20]	Predictive study	Nurses from three urban tertiary hospital *n* = 344	Nurse manager’s transformational leadership and nurse’s emotional intelligence are two predictors of workplace social capital	Describe determinants (block 1) and propose relational statements
Xu et al., 2020 [4]	Concept analysis	Twenty-six journal articles *n* = 26	The main attributes of nurses’ workplace social capital: relational network; trust; reciprocity; shared understanding; and social cohesion.	Facilitate the understanding of the constitution of the focal concept
Chang et al., 2019 [21]	Cross-sectional design	Nurses from one hospital *n* = 502	The positive relationship between workplace social capital and willingness to improve professional capabilities	Identify nurses’ outcomes and propose relational statements
Pham et al., 2019 [22]	Cross-sectional design	Nurses from a large medical center *n* = 166	Mentor–mentee rapport (social capital relationships) was positively related to the willingness to mentor/be mentored at work	Identify nurses’ outcomes and propose relational statements
Pittman et al., 2019 [23]	Descriptive correlational design	A large multisite healthcare system Nurses *n* = 701 Nurse managers *n* = 94	Hospital type, education, work role and employment status were the factors influencing workplace social capital	Describe determinants (block 2) and propose relational statements
Williamsson et al., 2019 [24]	Quantitative longitudinal study	Nurses from 20 units of five hospitals	Management tools use in nurses’ daily work positively modified workplace social capital	Describe determinants (block 1) and propose relational statements
Jafari et al., 2018 [25]	Cross-sectional study	Nurses from six public educational hospitals *n* = 215	Positively relationships between workplace social capital and clinical risk management	Identify organizational outcomes and propose relational statements
Middleton et al., 2018 [14]	Cross-sectional study (instrument validation)	Nurses who attended an upgrade degree program *n* = 362	Workplace social capital could relieve nurses’ mental distress and improve nurses’ health status	Identify nurses’ outcomes and propose relational statements
Norikoshi et al., 2018 [15]	Qualitative study	Nurses from 7 hospitals *n* = 32	Six groups of attributes of Japanese nurses’ workplace social capital: affirmation; exchange of appreciation; unrestricted information sharing; ability to trust; access to the strength; and altruistic reciprocity	Facilitate the understanding of the constitution of the focal concept
Tei-Tominaga & Nakanishi, 2018 [26]	Cross-sectional study	Nurse from 11 hospitals *n* = 822	Workplace social capital could result in less desirable outcomes such as exclusion of outsiders (social exclusion)	Identify nurses’ outcomes and propose relational statements
Gilbert et al., 2017 [17]	Model construction	Thirty-seven journal articles *n* = 37	Developed Gilbert Conceptual Model of Organizational Intellectual Capital in which workplace social capital plays an important role in nursing leadership	Facilitate the understanding of the constitution of the focal concept
Shin & Lee, 2017 [7]	Cross-sectional, correlational design	Nurses from two university-affiliated teaching hospitals *n* = 432	Positive relationships between workplace social capital and adoption of evidence-based practices	Identify nurses’ outcomes and propose relational statements
Strömgren et al., 2017 [27]	Longitudinal survey	Registered and assistant nurses from five hospitals *n* = 614	Overall leadership quality was positively related to workplace social capital	Describe determinants (block 1) and propose relational statements
Shin & Lee, 2016 [28]	Cross-sectional, correlational design	Nurses from two university-affiliated teaching hospitals *n* = 432	Workplace social capital varied based on different levels of education, years of experience and years in the present unit. Workplace social capital was positively associated with job satisfaction and quality of care	Describe determinants (block 2) and propose relational statements Identify nurses’ and patients’ outcomes and propose relational statements
Andersen et al., 2015 [29]	Cluster randomized controlled trial	Nurses and nurse’s aides from 18 departments at three hospitals *n* = 200	Group-based physical exercise could improve workplace social capital	Describe determinants (block 1) and propose relational statements
Read & Laschinger, 2015 [30]	Longitudinal survey	New graduate nurses *n* = 191	Authentic leadership positively associated with workplace social capital	Describe determinants (block 1) and propose relational statements
Laschinger et al., 2014 [13]	Cross-sectional survey	Nurses from 25 acute care hospitals *n* = 525	Workplace social capital was positively related to a better quality of care and unit effectiveness	Identify patients’ and organizational outcomes and propose relational statements
Read, 2014 [12]	Concept analysis	Seven journal articles and one book chapter	Main attributes of workplace social capital: networks of social relationships at work; shared assets; shared ways of knowing and being	Facilitate the understanding of the constitution of the focal concept
Hofmeyer, 2013 [16]	Model (framework) construction	Literature and qualitative data	Developed the Social Capital Framework to enhance team relationships	Facilitate the understanding of the constitution of the focal concept
Sheingold & Sheingold, 2013 [31]	Instrument development (cross-sectional survey for field testing)	Nurses from six hospitals *n* = 325	Workplace social capital had a significant impact on job satisfaction and intention to stay	Identify nurses’ and patients’ outcomes and propose relational statements
Van Bogaert et al., 2013 [32]	Cross-sectional survey	Nurses from 8 hospitals *n* = 1201	Unit level nurse management had a significant impact on workplace social capital	Describe determinants (block 1) and propose relational statements
Chang et al., 2012 [33]	Cross-sectional study	Nurses from a major medical center *n* = 797	Workplace social capital had a positive effect on nurses’ knowledge sharing and patient safety	Identify nurses’ and patients’ outcomes and propose relational statements
Vardaman et al., 2012 [34]	Qualitative study	Interview data of nurses, nurse managers, and physicians (*n* = 80); observation of nursing activities; documents	Positive effects of using a communication tool on building workplace social capital	Describe determinants (block 1) and propose relational statements
Hsu et al., 2011 [35]	Cross-sectional survey	Nurses from a large medical center *n* = 797	Workplace social capital positively influenced organizational commitment	Identify nurses’ outcomes and propose relational statements
Kowalski et al., 2010 [36]	Cross-sectional survey	Nurses from four hospitals *n* = 959	Workplace social capital negatively affected emotional exhaustion and burnout	Identify nurses’ outcomes and propose relational statements
Ernstmann et al., 2009 [37]	Cross-sectional survey	Nurses from four hospitals *n* = 959	Positive associations between workplace social capital and clinical risk management	Identify organizational’ outcomes and propose relational statements
Dicicco-Bloom et al., 2007 [1]	Model construction	Literature; an exemplar case	Developed a model of social capital to enhance the relationships in primary care work environment	Facilitate the understanding of the constitution of the focal concept

* All the studies have been carefully read to understand the constitution of the focal concept of nurses’ workplace social capital (block 3)

## Results

Assumptions are beliefs about a phenomenon or an event acting as a premise to understand a theory [5]. The four assumptions that were the pillars of our work in knowledge synthesizing and model construction of the nurses’ workplace social capital theory are: 1) The essentiality of and the necessity for a comprehensive understanding of this phenomenon before interventions; 2) The necessity of a comprehensive understanding of the constitution of nurses’ workplace social capital itself, the potential determinants for its occurrence and the ensuing outcomes; 3) A conceptual model with a graphic display, supported by empirical evidences, can help to produce a compact representation of a phenomenon which could enable to form a framework for future investigations and practical applications; and 4) The necessity for a continuous evaluation of nurses’ workplace social capital to propel the evolution of the model in response to the rapid changes in the profession of nursing.

### The focal concept of the conceptual model

Focal concept(s) specification is the first step in the process of theory synthesis. The focal concept of “nurses’ workplace social capital” was specified as the beginning of developing our conceptual model. We justified our approach because the workplace social capital increasingly has gained traction and importance in influencing work-life of the nursing profession.

When examining the constitution of main concept of nurses’ workplace social capital, the focus of attributes and classifications (component, type, and level) emerged. Two groups of attributes, relational networks, and the assets (e.g., trust, reciprocity, shared understanding, social cohesion), embedded in these networks, are the key characteristics of social capital. The attribute of relational network indicates the “doing” among people (structural component) who are weaving the fabric of workplace social capital, whereas the assets suggest the “feeling” among them (cognitive component) [2, 38, 39]. These theoretical notions have been adopted by researchers in the field of academic nursing, indicating their validity and applicability within the concept of nurses’ workplace social capital [4, 13, 16, 31, 40]. The notion of a three-component workplace social capital (structural, relational and cognitive), suggested by the work of Nahapiet and Ghoshal [11], has been applied in some nursing studies [1, 30, 33]; however, we have opted for the two-component construction of workplace social capital, because, relational social capital (e.g. the assets of trust, reciprocity) can be classified into cognitive component [4].

**Fig. 1:**
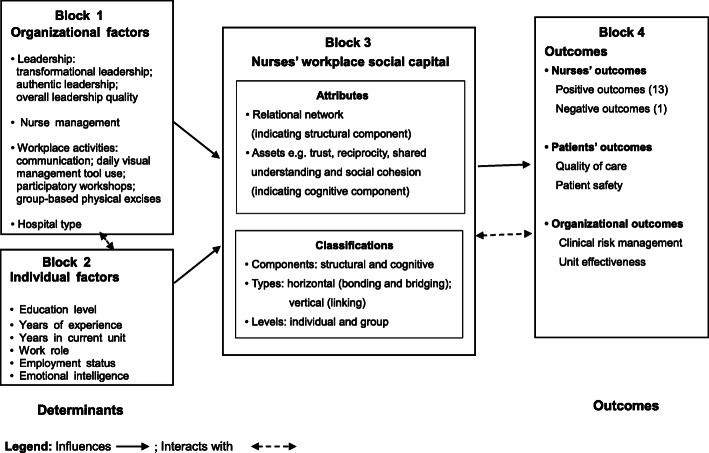
Conceptual model of nurses’ workplace social capital

Nurses’ workplace social capital can be classified into three types, bonding (relationships between nurses), bridging (e.g., relationships between nurses and other healthcare staff), and linking (e.g., relationships between nurses and head nurses) [4, 14, 16, 19, 29, 31]. Bonding and bridging describe relationships established within and/or among groups at the same professional and power level and, therefore, is regarded as horizontal social capital; in contrast, linking social capital represents relationships across different strata of power and is considered vertical social capital. The distinction of these three types of social capital enables different access and participation of a relational network at the workplace to be examined [13]. Finally, nurses’ workplace social capital can be organized into two levels, individual and group. Individual social capital refers to the micro relational networks around a person; while, group social capital is the macro networks woven by the intertwined relationships within a workgroup or with others outside [4, 36, 37]. The diagram of the constitution of this focal concept is depicted in Fig. 1 and complementary definitions are specified in Table 2.

**Table 2:** Definitions of main concepts in the model of nurses’ workplace social capital

Concept	Definition
**Determinants**	Factors that can influence the development of nurses’ workplace social capital, including, but not limited to, the two summary concepts - organizational factors and nurses’ individual factors, which were generated from the less general concepts from empirical evidence.
Organizational factors	Influencing factors related to organizations, an umbrella term to capture the less general concepts of leadership, nurse management, workplace activities and hospital type.
Individual factors	Influencing factors relevant to individual nurses grouped by the less general concepts of education level, years of experience, years in current unit, work role, employment status and emotional intelligence.
**Nurses’ workplace social capital**	“A relational network configured by reciprocated respectful interactions among nursing professionals and between the other healthcare professionals. These interactions are characterized by the norms of trust, reciprocity, shared understanding and social cohesion” [4]. It consists of two components (structural and cognitive) and three types (bonding, bridging and linking).
Structural social capital	The structure of social capital (what people do; the extent and intensity of their social interactions in the relational network).
Cognitive social capital	The assets embedded in and mobilized by the relational structure (what people feel: e.g., trust, reciprocity, shared understanding, social cohesion).
Bonding social capital	The relationships among people with similar positions and functions at work (nurses to nurses).
Bridging social capital	The relationships between people with different positions and functions at work (e.g. nurses to physicians, receptions and other staff).
Linking social capital	The relationship between people who are at different hierarchical level (e.g., nurses to head nurses).
Vertical social capital	Same as linking social capital
Horizontal social capital	The sum of bonding and bridging social capital
Individual social capital	The micro relational networks around a person
Group social capital	The macro relational networks wove by intertwined relationships in a workgroup or with others outside.
**Outcomes**	Results of the development of nurses’ workplace social capital, incorporating three summary themes - nurses’ outcomes, patients’ outcomes and organizational outcomes which are collapsed cross less general variables from empirical studies.
Nurses’ outcomes	Results related to nurses which include more concrete positive outcomes (e.g. increase of job satisfaction, professional commitment) and one negative outcome (social exclusion).
Patients’ outcomes	Results relevant to patients: the increase of quality of care and patient safety.
Organizational outcomes	Results pertinent to healthcare organizations: the improvement of clinical risk management and unit effectiveness in healthcare organizations.

### Related factors and relational statements in the conceptual model

The second step in the process of theory synthesis is to identify factors that are related to the focal concept and to analyze how these factors influence each other; while the third step is to logically organize all the concepts and statements in a diagrammatic form [6]. The ideas of “inventory of determinants or results” and “theoretical blocks” are the underpinning principles of these two processes [6, 41].

We also benefitted from Miller’s theory of human thought and cognition [42] in the second step of our theory synthesis. The Miller’s theory suggests that emerging cognitive events (such as nurses’ workplace social capital in this case), arise when certain existing inputs (determinants) lead to outcomes. Social capital is the subjective perceptions of individuals about their relationships with others at work; in other words, social capital can be interpreted as a conglomerate of the complex interactions among our thoughts, perceptions, and cognitions about our work environment. Therefore, we have arranged all the influencing factors on social capital under the themes of inputs (determinants), events (nurses’ workplace social capital) and outcomes and have specified their relationships (Fig. 1).

Accordingly, we developed a template to record the summarized empirical evidence in which the “Focal Concept (event)” was set in the middle column, while “Inputs” (determinant) and “Outcomes” were placed into the left and right columns, respectively. We identified the related concepts by reviewing the selected literature and then classified these concepts under the categories of Event, Inputs or Outcomes, per their content meaning and conveyed membership. Furthermore, similar but less general, sub-concepts were collapsed into more comprehensive summary concepts to reach the parsimony of the newly synthesized model. For example, different types of leadership and overall leadership quality were categorized under the summary concept of “leadership”. This summarized concept then was grouped under the high-order concept of “organizational factors” along with sub-concepts of “nurse management”, “workplace activities” and “hospital type”. Similarly, relationships between inputs/outcomes and the focal concept were collapsed and classified to higher-order relational statements. Five major relational statements, illustrating the relationships among these related concepts and the focal concept, were proposed for our conceptual model. These related concepts and relational statements are discussed below.

### Determinants of nurses’ workplace social capital and their relationships

*The first recommended relational statement in our proposed conceptual model is the organizational factors that influence the development of nurses’ workplace social capital.* In the nursing literature, two types of leadership have been described as the determinants of nurses’ workplace social capital: 1) Transformational leadership has been recognized as a strong predictor of nurses’ workplace social capital [20]; 2) Authentic leadership has been identified as a significant influencer of workplace social capital [30]. Additionally, research suggests that overall leadership quality significantly influences workplace social capital over time [27]. Amicable and situation-responsive nurse management at a unit has a positive and chronic influence on the development of workplace social capital [32].

Nurses’ workplace social capital is influenced by workplace behaviors and/or activities. We would like to use the term “Effect Modifiers” to describe the variables that influence, either negatively or positively, the nurses’ workplace social capital. For example, communication can be classified as an effect modifier of the nurses’ workplace social capital; poor quality and ineffective communication at work can quickly destroy nurses’ workplace social capital [13]. The style of communication, which endorses understanding and effective comprehension of messages, can strengthen the nurses’ workplace social capital. The impact of constructive communication, as a positive effect modifier, was reported by Vardaman et al. [34]. The authors reported on the long-term positive effects of the communication tool, Situation-Background-Assessment-Recommendation (SBAR) on nurses’ workplace social capital [34].

The spectrum of effect modifiers of nurses’ workplace social capital is broad and not exclusive to communication. For example, visual management tools in nurses’ daily work have been reported to positively modify nurses’ workplace social capital [24]; research has supported the positive effects of the organizational intervention of participatory workshops on the topic of utilizing assistive devices in patient handling, or group-based physical exercise on nurses’ workplace social capital [19, 29]. Finally, urgency, efficiency, and immediacy of delivery of healthcare services can be viewed as a positive effect modifier on nurses’ workplace social capital. Research supports the notion of higher workplace social capital among nursing professionals working in critical care hospitals compared with those working in community or academic hospitals [23].

*The second recommended relational statement in our proposed conceptual model is the individual factors that influence the development of nurses’ workplace social capital.*

Shin and Lee [28] reported that the score of workplace social capital varied among nurses’ groups with different levels of education, years of experience and years in the present unit. The scores of workplace social capital perceived by nurses with a graduate degree, providing direct care (work role) and having full-time employment status were lower than those who had bachelor’s education, provided non-direct care, and had part-time/casual work employment [23]. Moreover, employees with higher emotional intelligence are more dexterous in establishing constructive communication [43], in their interactions with others [44] and in developing interpersonal relational networks [45]. The positive influence of emotional intelligence on workplace social capital has been confirmed in the nursing population [20].

### Outcomes of nurses’ workplace social capital and their relationships

Eighteen outcomes, 17 positive and one negative, were identified in the nursing literature. These outcomes were then collapsed under the summary concepts to reach theoretical succinctness. Three summary concepts were abstracted from the more concrete outcomes: nurses’ outcomes (positive and negative), patients’ outcomes and organizational outcomes (Fig. 1).

*The third relational statement of the conceptual model is nurses’ outcomes, which is influenced by the nurses’ workplace social capital.* The less general concepts, under the summary term “nurses’ outcomes”, are 13 positive and one negative outcomes. The 13 positive nurses’ outcomes range from attenuation of emotional exhaustion, lower burnout and mental distress, increase in healthy self-behaviors, improvement in job satisfaction, strengthening the intention to stay, knowledge sharing, organizational commitment, professional commitment, motivation to improve professional capabilities, willingness to mentor/be mentored, adoption of evidence-based practice and prevention of occupational injuries and accidents; while, social exclusion is the only negative outcome of nurses’ workplace social capital.

Nurses’ workplace social capital is negatively related to emotional exhaustion and burnout [36]. Additionally, it may relieve nurses’ mental distress and can improve nurses’ health status [16]. Furthermore, nurses’ workplace social capital is positively associated with job satisfaction [28, 31] and intention to stay [31]. Nurses who perceive higher workplace social capital are more likely to share their knowledge with others [33] and develop higher organizational and professional commitments [21, 35]. Meanwhile, they have the willingness to improve their professional capabilities [21], mentor/be mentored at work [22] and adopt evidence-based practices [7]. Finally, workplace social capital is described by nurses as a major strategy for prevention of occupational injuries and accidents [3]. However, workplace social capital is also reported to result in social exclusion; strong bonding among the nursing staff can create strong relational ties that may influence their acceptability of newcomers [4, 14, 26].

*The fourth relational statement is patients’ outcomes which is influenced by the nurses’ workplace social capital.* “Patients’ outcomes” is a summary of two sub-concepts. First, a higher nurses’ workplace social capital leads to a better quality and more efficient delivery of care [15, 28]. Second, nurses’ self-report of patient safety also is indicative of the positive impacts of high nurses’ workplace social capital on patients’ outcomes [33].

*The fifth relational statement is organizational outcomes* which is influenced by *nurses’ workplace social capital.* Under the summary concept of “organizational outcomes” we have listed two distinct outcomes, better clinical risk management and improved unit effectiveness. Nurses’ workplace social capital is positively correlated with the betterment of clinical risk management [25, 37]; also, improved unit effectiveness, which has been defined as the capability of a unit to effectively and timely provide healthcare services, is positively correlated with the nurses’ workplace social capital [15].

### An integrated representation of the conceptual model

Finally, all the related concepts and relational statements were integrated into four “theoretical blocks” [6, 46] (Fig. 1) in our synthesized conceptual model with specific definitions of the main concepts (Table 2). This conceptual model illustrates the determinants, constitution and outcomes of nurses’ workplace social capital and specifies the relational statements among these concepts. Our conceptual model, with both graphic and narrative presentations, provides an updated and comprehensive information about nurses’ workplace social capital.

In our conceptual model, nurses’ workplace social capital (Block 3) is characterized with the attributes of relational network and several assets, classified by components (structural and cognitive), types (horizontal: bonding and bridging; vertical: linking) and levels (individual and group). Also, nurses’ workplace social capital may be influenced by both organizational factors (Block 1) and nurses’ individual factors (Block 2). In our model, organizational factors include leadership (transformational leadership, authentic leadership and leadership quality), nurse management, workplace activities (communication, daily visual management tool use, participatory workshops and group-based physical exercise) and hospital type, while nurses’ individual factors comprise the less general concepts of educational level, years of experience, years in current unit, work role, employment status and emotional intelligence. We also have demonstrated the interactions between these two categories of determinants, marked by a double arrow line in the model. These interactions are indicatives of the mutual supplementary effects of organizational factors and individual factors.

Eighteen variables were identified as the less general outcomes; these variables have been classified under three themes: nurses’ outcomes, patients’ outcomes, and organizational outcomes (Block 4). The improvements in nurses’ workplace social capital can lead to 17 positive outcomes. However, the strengthening of bonding social capital may lead to social exclusion.

We have demonstrated the possible interactive relationships among these variables; we emphasize the term “possible” because most of the outcomes were identified from cross-sectional studies which have limitations in discerning the symmetry (direction) of a statement [21, 22, 25, 28, 30]. Future prospective studies can either support or refute our proposed model.

## Discussion

We conducted a theoretical synthesis by Walker and Avant [6] and developed a theoretical model to amalgamate the current separated knowledge about nurses’ workplace social capital pillared by four theoretical assumptions. Our conceptual model depicts the constitution (attributes and classification), determinants (organizational and individual factors summarized from lower-level variables) and outcomes relevant to nurses, patients, and organizations. These outcomes have been collapsed into 18 less general outcomes and arranged into four distinct but interrelated blocks. Finally, the proposed five propositions among these blocks are supported by research evidence [1, 3, 4, 7, 15, 16, 19–37].

Nursing is a distinct science and nursing is a sovereign healthcare profession. It behooves the nursing profession and the nursing professionals to derive theories from the nursing domain [5, 6, 47]. Our conceptual model was synthesized on the premises of the nursing science and literature which contrasts with the previously developed theoretical models [1, 13, 14]. For example, the Social Capital Model by DiCicco-Bloom et al. [1], or the Social Capital Framework by Hofmeyer [13] or Gilbert Conceptual Model of Organizational Intellectual Capital [14] were developed based on literature sources from professions outside nursing or incorporated limited nursing research evidence. Application of these models in the nursing work environment might be hindered due to these limitations [14].

Our model was founded on previously developed models by nursing scholars [15, 30, 32]. These models were developed by applying statistical procedures such as path analysis or structural equation modeling to investigate the relationships between social capital and several independent variables and effect modifiers; the pertinent results of these studies have been used as the support for the current theory synthesis. To our knowledge, this newly synthesized model is the first conceptual model grounded in the integrative nursing evidence and follows the logical approach of theory synthesis [6].

A conceptual model can be applied in research, practice, and teaching in nursing science [5, 6, 14]. This synthesized model may point some clues for further research; for example, other potential determinants may be explored through both quantitative and qualitative investigations, especially for nurses’ individual factors. Or new concepts and statements could be entered into the conceptual model with further development of this theory and accumulation of more evidences. The other venues for advancing research in this arena include: 1) Assessment of interactional effects of the two categories of determinants on the focal concept. 2) Further assessment of the mediators between the determinants and nurses’ workplace social capital; more attention may be added to mediators and moderators between related variables and nurses’ workplace social capital; and 3) Implementation of longitudinal and interventional research to confirm the causal relationships among the focal concepts and outcomes.

Our conceptual model also can be applied in nursing practice. When seeking to improve the relevant outcomes in the healthcare organization, nursing administrators could try to achieve their goals through interventional programs on nurses’ workplace social capital with consideration of the determinants listed in this model. Meanwhile, the compromising outcome of social exclusion among the nursing workforce should be noted. However, an appropriate level of bonding may increase “togetherness” among group members [38]. The balance between restricting and formulating bonding social capital among nurses needs further exploration [4]. Additionally, our model also is conducive to teaching programs for nursing students and clinical nurses. The concept of workplace social capital is a subjective and abstract concept [4, 12, 14]; therefore, it is not easy to illustrate and understand its whole realm which involves the constitution of the construct, its related concepts, and the interrelationships among them. Our proposed summarized conceptual model with graphic and linguistic presentations makes the process of teaching and learning more manageable.

The process of developing our conceptual model has some limitations. First, the literature search was restricted to English language, peer-reviewed journal articles. Thereby, some information about nurses’ workplace social capital could have been missed. Second, the hypothesized interactional relationships (marked by the double arrow line in the model) need empirical confirmation. Despite the limitations of our manuscript, our work is the segue to future research and new findings and could provide new insights into the theory construction of nurses’ workplace social capital.

## Conclusion

In recent years, the precipitous attention of nursing scholars and researchers to nurses’ workplace social capital has made it necessary to capture a comprehensive insight into this phenomenon. Our newly synthesized conceptual model provides an effective way of approaching this goal. The strategy used for developing our conceptual model of nurses’ workplace social capital is the theory synthesis proposed and developed by Walker and Avant [6]. Our proposed model can be used as a foundation for further research based on identified gaps of current knowledge in the literature and the proposed propositions. Nursing practices that aim to strengthen nurses’ workplace social capital can consider the identified determinants. More studies are required to continuously enrich the current pool of evidences to address complexity of this conceptual model [6]. New knowledge should be integrated into our proposed model based on the evidence from model testing and the expansion of empirical investigations.

## Data Availability

Data sharing is not applicable to this article as no datasets were generated or analyzed during the current study.

## Abbreviations

CINAHL: Cumulative index to nursing and allied health literature
MeSH: Medical subject headings
